# In-Plane Gradient Magnetic Field-Induced Topological Defects in Rotating Spin-1 Bose–Einstein Condensates with SU(3) Spin-Orbit Coupling

**DOI:** 10.3390/e27050508

**Published:** 2025-05-09

**Authors:** Hui Yang, Peng-Yu Li, Bo Yu

**Affiliations:** 1 Department of Physics, Xinzhou Normal University, Xinzhou 034000, China; 2 College of Information Science and Engineering, Northeastern University, Shenyang 110819, China

**Keywords:** topological defects, in-plane gradient magnetic field, SU(3) SOC, spin *F* = 1 Bose–Einstein condensates

## Abstract

We study the topological defects and spin structures of rotating SU(3) spin–orbit-coupled spin *F*=1 Bose–Einstein condensates (BECs) in an in-plane quadrupole field with ferromagnetic spin interaction, and the BECs is confined by a harmonic trap. Without rotation, as the quadrupole field strength is increased, the spin *F*=1 BECs with SU(3) spin–orbit coupling (SOC) evolves from the initial Thomas–Fermi phase into the stripe phase; then, it enters a vortex–antivortex cluster state and eventually a polar-core vortex state. In the absence of rotation with the given quadrupole field, the enhancing SU(3) SOC strength can cause a phase transition from a central Mermin–Ho vortex to a vortex–antivortex cluster, subsequently converting to a bending vortex–antivortex chain. In addition, when considering rotation, it is found that this system generates the following five typical quantum phases: a three-vortex-chain cluster structure with mutual angles of approximately $\frac{2\pi }{3}$, a tree-fork-like vortex chain cluster, a rotationally symmetric vortex necklace, a diagonal vortex chain cluster, and a density hole vortex cluster. Particularly, the system exhibits unusual topological structures and spin textures, such as a bending half-skyrmion–half-antiskyrmion (meron–antimeron) chain, three half-skyrmion (meron) chains with mutual angles of an approximately $\frac{2\pi }{3}$, slightly curved diagonal half-skyrmion (meron) cluster lattice, a skyrmion–half-skyrmion (skyrmion-meron) necklace, and a tree-fork-like half-skyrmion (meron) chain cluster lattice.

## 1. Introduction

In recent years, spin–orbit-coupled quantum gases have been the subject of great interest, both in experimental and theoretical studies [1,2,3,4,5,6]. In particular, the spin–orbit-coupled Bose–Einstein condensates (BECs) provide a brand-new platform to investigate the novel spin–orbit coupling (SOC) physics and prospective applications because of their ultrahigh purity, precise experimental controllability, and excellent theoretical description [7,8,9]. The SOC between two hyperfine states in ultracold atoms significantly alters the single-particle dispersion. To date, numerous investigations have revealed that the competition between the SOC and rotation in two-component BECs can not only result in rich quantum states, for example, supersolid state [10], various stripe phases [11,12], soliton generation [13], and quantum beating [11,14], but they can also stabilize various topological excitations of the BECs, such as the half-quantum vortex [15,16], vortex necklace [11,17], skyrmion [17,18], skyrmion string [11], skyrmion-meron lattice, and Bloch domain wall [16]. Most of these studies were related with the SU(2)-type SOC, where the internal states couple to their momentum via the SU(2) Pauli matrices. Recently, the spinor BECs with more spin degrees of freedom have attracted much attention, and the spin-1 BECs with SU(2) SOC has been realized experimentally [19]. However, for spin-1 BECs, if there exists coupling between any two internal states of the three-component system, the SU(3) SOC will be more effective than the SU(2) SOC as the spin operator, the former case spanned by Gell-Mann matrices [20,21,22,23]. The system, featuring SU(3) SOC, is expected to give rise to novel quantum phenomena and intriguing properties. For spin-1 BECs with antiferromagnetic interaction, the SU(3) may give rise to the formation of double-quantum spin vortices [21].

In addition, gradient magnetic fields were employed to realize some novel quantum states and quantum phase transitions [24,25,26]. Related studies show that SOC and the spin Hall state can be realized in an optical lattice system by applying a gradient magnetic field [27]. The above studies indicate that gradient magnetic fields play a crucial role in the generation of artificial gauge fields and new topological excitations. Therefore, it will be interesting to investigate the topological excitations and nontrivial quantum phases of spinor BECs by combining the gradient magnetic field, SU(3) SOC, and other important experimental parameters.

In this work, we investigate the joint effects of SU(3) SOC, the in-plane quadrupole field, and rotation on the ground-state structures of spin-1 BECs with ferromagnetic spin interaction. We discover that this system sustains varied topological excitations. We analyze two cases of the system in the absence of rotation and in the presence of rotation, respectively. For the former case, the system sustains a Thomas–Fermi phase, stripe phase, vortex–antivortex cluster state, a polar-core vortex state, a central Mermin–Ho vortex, vortex–antivortex cluster, and a bending vortex–antivortex chain, depending on the quadrupole field, SU(3) SOC. Particularly, for the latter case (i.e., the rotation case), this system gives rise to five representative quantum phases as follows: three-vortex-chain cluster structures with mutual angles of approximately $\frac{2\pi }{3}$, a tree-fork-like vortex chain cluster, a rotationally symmetric vortex necklace, a diagonal vortex chain cluster, and a density hole vortex cluster. In addition, this system sustains exotic spin textures and skyrmion configurations, including a bending half-skyrmion–half-antiskyrmion (meron–antimeron) chain, three half-skyrmion (meron) chains with mutual angles of approximately $\frac{2\pi }{3}$, a slightly curved diagonal half-skyrmion (meron) cluster lattice, a skyrmion–half-skyrmion (skyrmion-meron) necklace, and a tree-fork-like half-skyrmion (meron) chain cluster lattice.

The following sections are organized as follows. In Section 2, the theoretical model is described. In Section 3, we analyze and discuss the ground states, the corresponding spin textures, and skyrmion configurations. We summarize the main findings in Section 4.

## 2. Model

By considering strong confinement in the *z*-direction, we investigate a two-dimensional SU(3) spin–orbit-coupled *F*=1 BECs in a rotating harmonic trap with in-plane gradient magnetic field. In the mean-field approximation, the system’s energy can be expressed as [21,25,28].

(1) $$\begin{array}{ccc} E & = & \int dr\sum_{m}\Psi_{m}^{*}\left[\left(-\frac{\hbar^{2}\nabla^{2}}{2M}+V\left(r\right)-\Omega L_{z}+g_{F}\mu_{B}B\left(r\right)\cdot f\right)\Psi_{m}\right]\\ & & +E_{so}+E_{sw}, \end{array}$$

where $\Psi_{m}\left(m=1,0,-1\right)$ is the component wave function. The total particle density is $n\left(r\right)=\sum_{m}{\left|\Psi_{m}\left(r\right)\right|}^{2}$, and *M* presents the atomic mass. The two-dimensional harmonic trap is $V\left(r\right)=m\omega^{2}\left(x^{2}+y^{2}\right)/2$, where ω and $a_{h}=\sqrt{\hbar /m\omega }$ are the radial trapping frequency and harmonic-oscillator length, respectively. Ω represents the rotation frequency in the *z*-direction, where Ω=0 represent no rotation and Ω>0 corresponds to the rotating case. $L_{z}=i\hbar \left(y\partial_{x}-x\partial_{y}\right)$ represents the *z* component of the angular-momentum operator. $g_{F}=-1/2$ is the Lande factor, and $\mu_{B}$ presents the Bohr magnetic moment. The in-plane gradient magnetic field (i.e., the in-plane quadrupole field) B(r) is expressed by $B\left(r\right)=B\left(xe_{x}-ye_{y}\right)$, with *B* being the strength of gradient magnetic field. $E_{so}$ and $E_{sw}$ are the SU(3) SOC energy and the short-range *s*-wave interaction energy, respectively. The SU(3) SOC energy is given by $E_{so}=\int dr\sum_{m,m^{\prime }}\Psi_{m}^{*}{\left(v_{so}\right)}_{mm^{\prime }}\Psi_{m^{\prime }}$, where the SU(3) SOC $v_{so}=k\left(\lambda_{x}p_{x}+\lambda_{y}p_{y}\right)$, *k* characterizes the SU(3)SOC strength, and $p_{x}$ and $p_{y}$ are two-dimensional momenta. $\lambda_{x}=\lambda^{\left(1\right)}+\lambda^{\left(4\right)}+\lambda^{\left(6\right)}=\left(\begin{array}{ccc} 0 & 1 & 1\\ 1 & 0 & 1\\ 1 & 1 & 0 \end{array}\right)$, and $\lambda_{y}=\lambda^{\left(2\right)}-\lambda^{\left(5\right)}+\lambda^{\left(7\right)}=\left(\begin{array}{ccc} 0 & -i & i\\ i & 0 & -i\\ -i & i & 0 \end{array}\right)$, where $\lambda^{\left(i\right)}\left(i=1,\cdots 8\right)$ are the Gell-Mann matrices, i.e., the generators of the SU(3) group [21,29]. The *s*-wave interaction energy can be described as

(2) $$E_{sw}=\int dr\left(\frac{c_{0}}{2}n^{2}+\frac{c_{2}}{2}{\left|F\left(r\right)\right|}^{2}\right),$$

where $c_{0}=4\pi \hbar^{2}\left(2a_{2}+a_{0}\right)/3M$ and $c_{2}=4\pi \hbar^{2}\left(a_{2}-a_{0}\right)/3M$ are the strengths of density–density and spin–exchange interactions [17], respectively, and $a_{s}\left(s=0,2\right)$ is the *s*-wave scattering length corresponding to the scattering channel with total spin. Generally, $c_{2}$ >0 represents antiferromagnetic interaction and $c_{2}$ <0 denotes ferromagnetic interaction.

For the case of SU(3) SOC, the dimensionless GP equations for the system’s dynamics are given by

(3) $$\begin{array}{ccc} i\frac{\partial \psi_{1}}{\partial t} & = & \left[-\frac{1}{2}\nabla^{2}+V+i\Omega \left(x\partial_{y}-y\partial_{x}\right)+\lambda_{0}{\left|\psi \right|}^{2}+\lambda_{2}\left({\left|\psi_{1}\right|}^{2}+{\left|\psi_{0}\right|}^{2}-{\left|\psi_{-1}\right|}^{2}\right)\right]\psi_{1}\\ & & +\left[B\left(x+iy\right)+k\left(-i\partial_{x}-\partial_{y}\right)\right]\psi_{0}+k\left(-i\partial_{x}+\partial_{y}\right)\psi_{-1}+\lambda_{2}\psi_{-1}^{*}\psi_{0}^{2}, \end{array}$$

(4) $$\begin{array}{ccc} i\frac{\partial \psi_{0}}{\partial t} & = & \left[-\frac{1}{2}\nabla^{2}+V+i\Omega \left(x\partial_{y}-y\partial_{x}\right)+\lambda_{0}{\left|\psi \right|}^{2}+\lambda_{2}\left({\left|\psi_{1}\right|}^{2}+{\left|\psi_{-1}\right|}^{2}\right)\right]\psi_{0}\\ & & +\left[B\left(x-iy\right)+k\left(-i\partial_{x}+\partial_{y}\right)\right]\psi_{1}+\left[B\left(x+iy\right)+k\left(-i\partial_{x}-\partial_{y}\right)\right]\psi_{-1}+2\lambda_{2}\psi_{1}\psi_{-1}\psi_{0}^{*}, \end{array}$$

(5) $$\begin{array}{ccc} i\frac{\partial \psi_{-1}}{\partial t} & = & \left[-\frac{1}{2}\nabla^{2}+V+i\Omega \left(x\partial_{y}-y\partial_{x}\right)+\lambda_{0}{\left|\psi \right|}^{2}+\lambda_{2}\left({\left|\psi_{-1}\right|}^{2}+{\left|\psi_{0}\right|}^{2}-{\left|\psi_{1}\right|}^{2}\right)\right]\psi_{-1}\\ & & +\left[B\left(x-iy\right)+k\left(-i\partial_{x}+\partial_{y}\right)\right]\psi_{0}+k\left(-i\partial_{x}-\partial_{y}\right)\psi_{1}+\lambda_{2}\psi_{1}^{*}\psi_{0}^{2}. \end{array}$$

with the dimensionless wave function for the *j*-th component $\psi_{j}=N^{-1/2}a_{h}\Psi_{j}\left(j=0,\pm 1\right)$ and the total particle density being ${\left|\psi \right|}^{2}={\left|\psi_{1}\right|}^{2}+{\left|\psi_{0}\right|}^{2}+{\left|\psi_{-1}\right|}^{2}$. Here the external potential in dimensionless form is $V=(x^{2}+y^{2})/2$. $\lambda_{0}=4\pi N\left(2a_{2}+a_{0}\right)/3a_{h}$ and $\lambda_{2}=4\pi N\left(a_{2}-a_{0}\right)/3a_{h}$ denote the dimensionless interactions of density–density and spin–exchange. *B*, *k*, and Ω denote dimensionless quadrupole field strength, SU(3) SOC strength, and rotation frequency, respectively [17]. In the numerical computations presented in our paper, the time, length, magnetic field gradient, and energy (interaction, SOC, and rotation) are quantified in units of 1/ω, $\sqrt{\hbar /m\omega }$, $\hbar \omega /(g_{F}\mu_{B}a_{h})$, and ℏω, respectively. As shown in Equations (3) and (5), there are direct transitions between the states $|1\rangle$ and $|-1\rangle$ due to the SU(3) SOC. By employing the extensively used imaginary-time evolution algorithm, we are able to numerically resolve the GP Equations (3)–(5) and obtain the topological defects of this system.

For F=1 spinor BECs, the spin texture can be described by [30]

(6) $$S_{\alpha }=\sum_{m,n=0,\pm 1}\psi_{m}^{*}{\left(f_{\alpha }\right)}_{m,n}\psi_{n}/{\left|\psi \right|}^{2}\left(\alpha =x,y,z\right).$$

The spatial distribution of the system’s topological structure can be described in terms of the topological charge density

(7) $$q\left(r\right)=\frac{1}{4\pi }s\cdot \left(\frac{\partial s}{\partial x}\times \frac{\partial s}{\partial y}\right),$$

and that topological charge *Q* is defined by

(8) Q=∫q(r)dxdy,

where $s=S/\left|S\right|$, and the topological charge $\left|Q\right|$ remains invariant under the transformation $\left(S_{x}^{\prime },S_{y}^{\prime },S_{z}^{\prime }\right)=\left(S_{y},S_{x},S_{z}\right)$ [31].

## 3. Results and Discussion

Owing to the existence of the in-plane quadrupole field, SU(3) SOC, rotation, and ferromagnetic spin interaction, no analytical solution exists for the coupled GP Equations (3)–(5). In what follows, we use the imaginary-time propagation method (ITPM) based in the Peaceman–Rachford method (PRM) to numerically solve the two-dimensional GP Equations (3)–(5), aiming to obtain the ground states by minimizing the total energy of the system [32,33]. The fundamental concept of the PRM is to transform a two-dimensional problem into one-dimensional problems, which can then be readily extended to three-dimensional case. The ITPM, utilizing the PR approach, exhibits excellent convergence, remarkable stability, and high precision. The convergence and accuracy of the algorithm are tested using the virial theorem, which establishes strict relationships between kinetic and potential energy contributions. Additionally, the energy functional and wave functions in the system converge rapidly, providing a second test. The algorithm also yields consistent results for different trial wave functions, serving as further validation. All these tests have been confirmed in our numerical computations. A notable characteristic of this system is the presence of a vast number of free parameters, including the rotation, SU(3) SOC, quadrupole field, ferromagnetic spin interaction, the density–density interaction, and spin–exchange interaction. Owing to the competition among multiple parameters, this system can display diverse ground-state structures and unusual topological excitations. In our simulation, we take into account the ferromagnetic spin interaction, i.e., $\lambda_{2}<0$. The typical parameters of the density–density and spin–exchange interactions are chosen as $\lambda_{0}$ =6000 and $\lambda_{2}=-50$, respectively.

### 3.1. Ground-State Structures and Spin Texture for the Nonrotating Case

**Figure 1:**
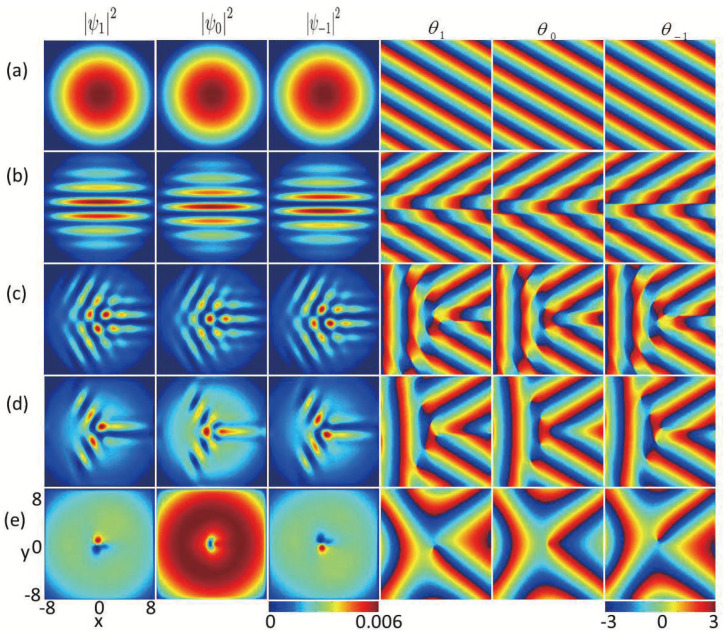
Density distributions and phase distributions for the ground states of SU(3) spin–orbit-coupled spin-1 BECs with an in-plane quadrupole field in a harmonic trap, where k=0.8, $\lambda_{0}=6000$ and $\lambda_{2}=-50$. The first three columns denote the density distributions of three components $|F=1,m_{F}\;=\;1\rangle$, $|F=1,m_{F}\;=0\rangle$, and $|F=1,m_{F}\;=-\;1\rangle$, and the corresponding phase distributions are displayed in the last three columns, respectively. These plots are for different strengths of in-plane quadrupole fields, with (**a**) B=0, (**b**) B=0.001, (**c**) B=0.1, (**d**) B=0.2, and (**e**) B=1.5.

Firstly, we analyze how the in-plane quadrupole field affects the ground state of ferromagnetic spin-1 BECs with fixed SU(3) SOC (k=0.8). In Figure 1, ${\left|\psi_{1}\right|}^{2}$, ${\left|\psi_{0}\right|}^{2}$ and ${\left|\psi_{-1}\right|}^{2}$ (three columns on the left side) denote the profiles of the density for the three components $m_{F}=1$, $m_{F}=0$, and $m_{F}-1$, and the relevant phase distributions are represented as $\theta_{1}=\arg\psi_{1}$, $\theta_{0}=\arg\psi_{0}$, and $\theta_{-1}=\arg\psi_{-1}$ (three columns on the right side), respectively. Without the in-plane quadrupole field strength (B=0), the system displays a completely mixed state and the maximum densities for the three components are centered at the harmonic trap, whereas the condensate’s phase fluctuates like a plane wave exp $\left(ik\cdot r\right)$, which is a Thomas–Fermi phase [34] in Figure 1a. For the very weak in-plane quadrupole field strength, i.e., B=0.001, the densities of the system display a spatially separated stripe state along the *x* direction, which is different from the stripe density patterns [35] as a result of the existence of the in-plane quadrupole field. For the latter case, the stripe density results from the combined of dipole–dipole interaction and SU(2) SOC. As the in-plane quadrupole field strength increases, e.g., B=0.1, a vortex–antivortex cluster consisting of vortices and antivortices in each component forms and a few ghost vortices [32,36] are distributed in the outskirts of the atom cloud (see Figure 1c). For the stronger in-plane quadrupole field (B=0.2) (see Figure 1d), there are two vortices (clockwise rotation) and three antivortices (anticlockwise rotation) in component $m_{F}$ =1, two vortices and two antivortices in component $m_{F}$ =0, and two antivortices and three vortices in component $m_{F}$ =−1, respectively. As shown from Figure 1c,d, the time-reversal symmetry is broken due to the joint influence of SU(3) SOC and the in-plane quadrupole field, which leads to the irregular distributions of phases for the three components. For a large quadrupole field strength, e.g., B=1.5, we find that the vortices far from the condensate center disappear (Figure 1e). The significant reduction in the number of vortices is a result of the reversal of magnetic moments derived from the in-plane magnetic fields. Here, the vortex configuration is characterized by the combination of winding numbers associated with every component. The configuration of windings can be written as $\langle w_{1},\;w_{0},\;w_{-1}\rangle$ with the integers $w_{1},\;w_{0},\;w_{-1}$ being the winding numbers of $\psi_{1},\;\psi_{0},\;\psi_{-1}$, respectively, and *w* represents the phase shifts by 2πw whenever the wave function circulates around the votex of the phase. We find that there are vortices in the core areas of components 1 and 3, and there are no vortice in the center of component 2, which gives rise to a polar-core vortex, in which the winding configuration is $\langle -1,0,1\rangle$ with an antiferromagnetic core, as shown in Figure 1c–e.

**Figure 2:**
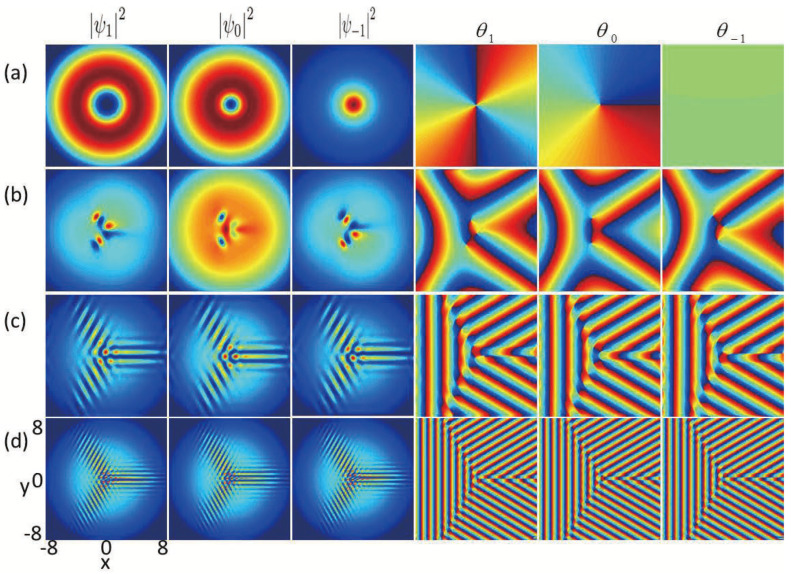
Density distributions and phase distributions for the ground states of SU(3) spin–orbit-coupled spin-1 BECs with an in-plane quadrupole field in a harmonic trap, where B=0.2, $\lambda_{0}=6000$, and $\lambda_{2}=-50$. The first three columns denote the density distributions of three components $|F=1,m_{F}\;=\;1\rangle$, $|F=1,m_{F}\;=0\rangle$ and $|F=1,m_{F}\;=-\;1\rangle$, and the corresponding phase distributions are displayed in last three columns, respectively. These plots are for different strengths of SU(3) SOC, with (**a**) k=0, (**b**) k=0.3, (**c**) k=1.5 (**d**) k=2.5.

Secondly, we study the effects of SU(3) SOC on the ground-state structures of nonrotating spin-1 BECs with the existence of an in-plane quadrupole field. The strength of the quadrupole field is set to B=0.2, while all other related variables remain consistent with those in Figure 1, except the SU(3) SOC strength. The primary findings are presented in Figure 2. As shown in Figure 2a, without SU(3) SOC (k=0), the winding numbers located in the middle of the three components $m_{F}$ =1, 0, −1 are given by $\langle -2,-1,0\rangle$. As a result, the vortex structure within this system belongs to a Mermin–Ho vortex [37] (Figure 2a), in which two quantized antivortices characterized by a winding number of −2 and a singly quantized antivortex characterized by winding number −1 are distributed in the middle of components $m_{F}$ =1 and $m_{F}$ =0, respectively, while a bright soliton characterized by a zero winding number forms in the central area of component $m_{F}$ =−1. When the SOC strength *k* is relatively small (e.g., k=0.3), there is a triangular vortex–antivortex lattice composed of a vortex and two antivortices in component $m_{F}$ =1, a vortex–antivortex pair in component $m_{F}$ =0, and a triangular vortex–antivortex lattice consisting of two vortices and an antivortex in component $m_{F}$ =−1, respectively. With the SU(3) SOC strength *k* increasing from 0.3 to 2.5, more phase defects form in each component and they develop into a vortex chain and an antivortex chain, respectively, at an angle of approximate 2π/3 as shown in Figure 2c,d. The transformations of vortex structures in Figure 2 are analogous to the topological phase transitions observed in nanomagnet systems [38]. Essentially, this intriguing feature results from the competition between the SU(3) SOC and the quadrupole field in spin-1 BECs and is inaccessible in other systems, where the SU(3) SOC term in the Hamiltonian involves all the pairwise coupling between the three spin states [20,21]. From Figure 1 and Figure 2, by introducing the in-plane quadrupole filed and SU(3) SOC as two new degrees of freedom, we can achieve the desired ground-state configurations and control the phase transition among different ground states of spin-1 BECs.

**Figure 3:**
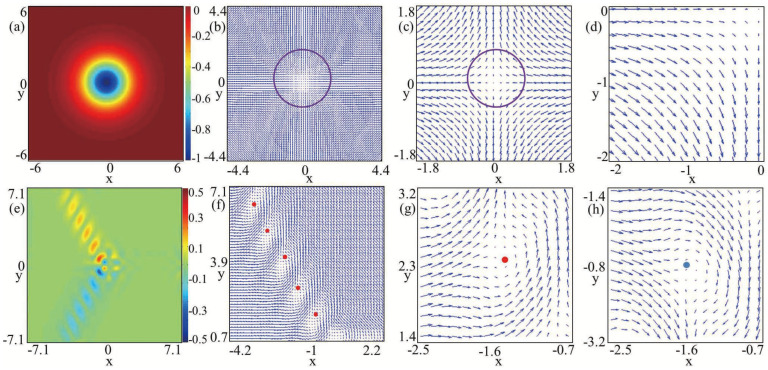
Topological charge densities and spin textures. (**a**–**d**) and (**e**–**h**) correspond to Figure 2a and Figure 2c, respectively. The left two columns denote the topological charge densities and spin texture, respectively. Furthermore, the right two columns represent the local amplifications of the spin texture.

Besides the line-like vortex excitation related to the spatial degrees of freedom of the BECs, the present system also supports topological excitations that are point-like in terms of the spin degrees of freedom, while skyrmions have already been observed in nuclear physics [39], and quantum Hall systems [40], creating these topological defects occurring ultracold gases could offer a novel platform for investigating their physical properties with higher precision. Moreover, owing to the exceptional purity and precise manipulability of the cold-atom system enable a thorough comparison between experimental observations and corresponding theories through skyrmion excitations in ultracold atomic gases. Combining SOC and rotation can result in a variety of topological excitations, such as circular skyrmion string [16], circular-hyperbolic skyrmion [18], giant skyrmion [41], hyperbolic half-skyrmion [42] and so on. Displayed in Figure 3a,b are the topological charge density and spin texture corresponding to the parameters presented in Figure 2a. The enlarged spin textures in the local region are given in Figure 3c,d. The spin defect depicted in Figure 3b is demonstrated to be an antiskyrmion characterized by a topological charge Q=−1. Recent studies have demonstrated that antiskyrmion in spin–orbit-coupled spin-1 BECs is Mermin–Ho vortex structure which can be written as ${\left(-2_{1},-1_{0},0_{-1}\right)}_{1}$, where, in the bracket, $-2_{1}$ presents the $m_{F}=1$ vortex characterized by a winding number −2, $-1_{0}$ denotes the $m_{F}=0$ vortex characterized by a winding number −1, $0_{-1}$ is component $m_{F}=-1$ vortex characterized by a winding number 0, and the subscript 1 placed outside the bracket signifies the overlapping of the three vortices.

By comparison, Figure 3e illustrates the topological charge density for nonrotating spin-1 BECs with SU(3) SOC, where B=0.2, k=1.5, and the ground state is exhibited in Figure 2c. Notice that the component densities in Figure 2c and the topological charge density in Figure 3e are both approximately symmetric (or antisymmetric) with respect to the y=0 axis. Considering the limited resolution of the texture, in Figure 3f, we only show the spin texture in the y>0 region, and the typical local amplifications of the full spin texture are given in Figure 3g and Figure 3h, respectively. Our numerical calculation shows that the red spots in Figure 3f,g denote half-skyrmions (merons) with localized topological charge Q=0.5 [18,37]. Simultaneously, the blue spots in Figure 3h indicate half-antiskyrmions possessing a localized topological charge of Q=−0.5. It is obvious that the spin defects in Figure 3f form a half-skyrmion (meron) chain. Therefore the full spin texture corresponding to the ground-state structures in Figure 2c consists of a half-skyrmion (meron) chain and a half-antiskyrmion (antimeron) chain, where the two chains form an angle of 2π/3. We call this special topological configuration a bending half-skyrmion–half-antiskyrmion (meron–antimeron) chain. Related studies have demonstrated that the half-skyrmion in quenched spin-1 BECs with SOC is associated with a three-vortex structure, which can be presented as ${\left(1_{1},1_{0},1_{-1}\right)}_{3}$ [18,43], i.e., each of the components $m_{F}=1,0,-1$ contains a vortex characterized by a winding number 1, and the subscript 3 outside the bracket indicates that the three vortices are located at three different positions. Thus, the structure composed of three vortices can be considered as a cell in which the ratio of vortices among the three components approaches 1:1:1.

### 3.2. Ground-State Structures and Spin Texture for the Rotating Case

**Figure 4:**
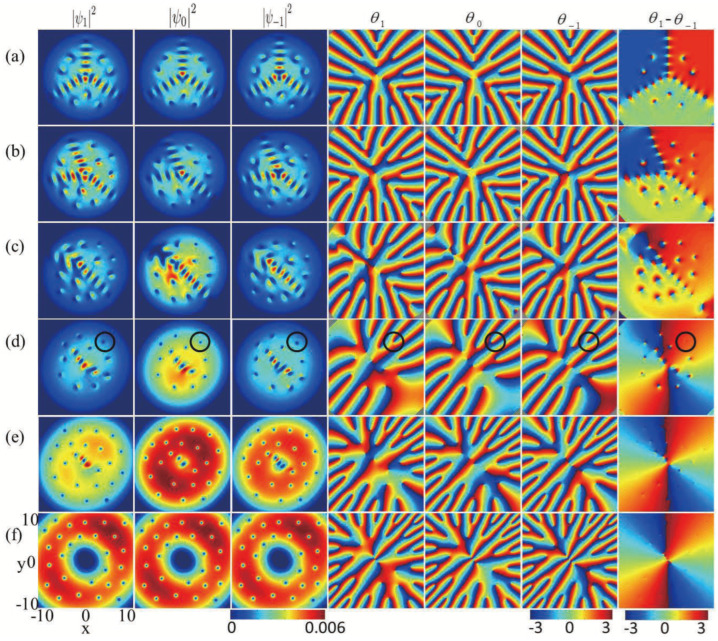
Effect of in-plane quadrupole fields on the ground-state structures of SU(3) spin–orbit-coupled spin-1 BECs with a fixed rotation Ω=0.3. The parameters are k=0.5, $\lambda_{0}$ =6000, and $\lambda_{2}=-50$. The first three columns denote the density distributions of the three components $|F=1,m_{F}\;=\;1\rangle$, $|F=1,m_{F}\;=0\rangle$, and $|F=1,m_{F}\;=-\;1\rangle$, and the corresponding phase distributions are given in the fourth to the sixth columns, respectively. The last column presents the phase difference between two components $m_{F}\;=\pm 1$. The strengths of the in-plane quadrupole field are given by (**a**) B=0, (**b**) B=0.1, (**c**) B=0.2, (**d**) B=1, (**e**) B=3, and (**f**) B=5.

Now, we study the ground states of rotating SU(3) spin–orbit-coupled spin-1 BECs in the presence of an in-plane quadrupole field. Firstly, we consider the role of the in-plane quadrupole field with fixed rotation frequency Ω=0.3. We begin with the case in which the quadrupole field is sufficiently weak (B=0). As shown in Figure 4a, the density distribution for every component consists of three vortex chains extending from the center of the condensate, with angles of approximately 2π/3 between any two chain. In addition, each of the three components is divided into three regions, which contain two vortices, two vortices, and three vortices, respectively. We call it a three-vortex-chain cluster structure. The phase difference presented in the final column of Figure 4a indicates that the phases are not synchronized in the components $m_{F}$ =±1, which differs from the cases of anisotropic SOC [11,18,43]. With the relatively weak quadrupole field (B=0.1), the angle between the two vortex chains, one aligned along the y-axis and the other in the third quadrant, decreases, while the vortex chain in the fourth quadrant remains unchanged. Meanwhile, the number of vortices in the regions divided by the vortex chains change, being one, four, and four, respectively. We can name this a tree-fork-like vortex chain cluster. With a further increase in the in-plane quadrupole field, e.g., B=0.2, the density distributions of three-vortex chains are destroyed due to the increased in-plane gradient magnetic field, and the vortices in individual components display diagonalized serpentine structures with some vortices distributed on both sides (see Figure 4c), which is different from the vortex chain phenomenon observed in antisotropic spin-1/2 BECs with SOC [11,18] and spin-1 BECs [17,43]. Here, a diagonalized serpentine chain is the result of the interaction between the in-plane quadrupole field, SU(3) SOC, and rotation.

Physically, the interaction between the in-plane quadrupole field and SU(3) SOC induces a bending vortex–antivortex chain, with the vortex chain located in the second quadrant and the antivortex chain in the third quadrant (see the right three columns of Figure 1c,d and those of Figure 2c,d). Meanwhile, the joint influence of the SU(3) SOC and rotation sustains a three-vortex-chain structure, located, respectively, along the *y* direction, in the third quadrant, and in the fourth quadrant, respectively (see Figure 4a). Hence, the collaborative results of the in-plane quadrupole field, SU(3) SOC, and rotation generate the diagonal serpentine vortex chain. We have calculated the phase difference between any two components $m_{F}$ =−1,0 and 1 in Figure 4, for instance, the phase differences between $m_{F}$ =−1 and $m_{F}$ =1 are displayed (the last column of Figure 4). The calculation results show that the phases are desynchronized in Figure 4a–c. Thus, all the vortices away from the vortex chain in the three components form the three-vortex configuration, differing from those in [43]. Here the interesting three-vortex configurations are caused by the interplay and competition among the in-plane quadrupole field, the isotropic SU(3) SOC, and the rotation. As the in-plane quadrupole field further increases (see Figure 4d–f), the vortices along the diagonal serpentine vortex chain decrease but those away from the diagonal serpentine vortex chain increase. By computing the phase differences between any two components $m_{F}$ =−1,0 and 1 (see the final column of Figure 4d–f), one can conclude that the phases gradually synchronize as the in-plane quadrupole field increases. Taking Figure 4d as an example, the outmost vortices of the three components in the second quadrant overlap (see the black circle of Figure 4d). For the case of B=5, by calculation, all vortices away from the density hole of the central region of the condensate overlap. The above can be explained as when the three vortices of the three components coincide, it will contribute more angular momentum and energy to the system. Therefore, the three vortices that overlap are more likely to appear in the low-density region of BECs (i.e., the boundary of BECs).

**Figure 5:**
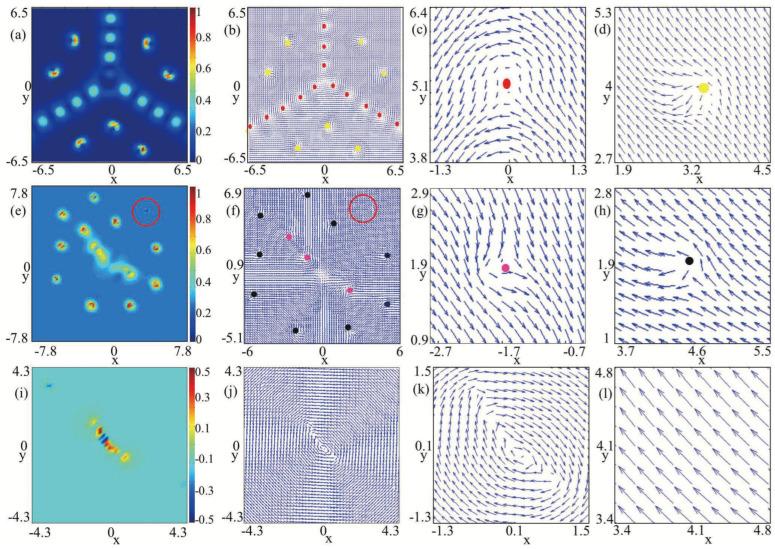
Topological charge densities and spin texture. (**a**–**d**), (**e**–**h**), and (**i**–**l**) correspond to Figure 4a, Figure 4d, and Figure 4f, respectively. The first column represents the topological charge densities, the second column denotes the spin textures, and the last two columns present the local amplifications of the spin texture.

Figure 5a,b illustrates the topological charge density and spin texture characteristics of rotating spin-1 BECs with SU(3) SOC, respectively. The corresponding ground state is displayed in Figure 4a. Notice that the topological charge density in Figure 5a and the component densities in Figure 4a are approximately symmetric about the x=0 axis. Figure 5c,d shows the prominent local magnifications of the spin texture in Figure 5b. By comparison, Figure 5e depicts the topological charge density for the SU(3) spin–orbit-coupled spin-1 BEC under rotation, where B=1, Ω=0.3, and k=0.5, and the ground state is exhibited in Figure 4d. The distinctive local amplifications of the entire spin texture of Figure 5f are given in Figure 5g and Figure 5h, respectively. Our numerical computation indicates that all the spots in Figure 5b–d,f–h denote half-skyrmions (merons) with local topological charge Q=0.5 [18,37]. Note that different colored dots in Figure 5 represent different shapes of spin textures corresponding to half-skyrmions, which result from the three-vortex structure. In addition, we find that the outermost vortices in the second quadrant of the three components did not form a clearly visible spin texture with topological density Q=0 (see the red circle of Figure 5e–f). It can be explained that the outermost vortices in the second quadrant of the three components overlap with the ${\left(1_{1},1_{0},1_{-1}\right)}_{1}$ vortex configuration, which aligns with the phase difference of Figure 4b.

The topological charge density and the spin texture are illustrated in Figure 5i,j, and corresponding density and phase distributions are shown in Figure 4f. Our numerical calculation shows that there is only an irregular elliptical shape half-skyrmion carrying topological charge Q=0.5 in the center of the condensate. Essentially, the above phenomenon is explained by the vortices away from the center of the condensate being overlapped in the three components. Therefore. these vortices can be written as ${\left(1_{1},1_{0},1_{-1}\right)}_{1}$, where the subscript 1 placed outside the bracket signifies that the three vortices coincide.

**Figure 6:**
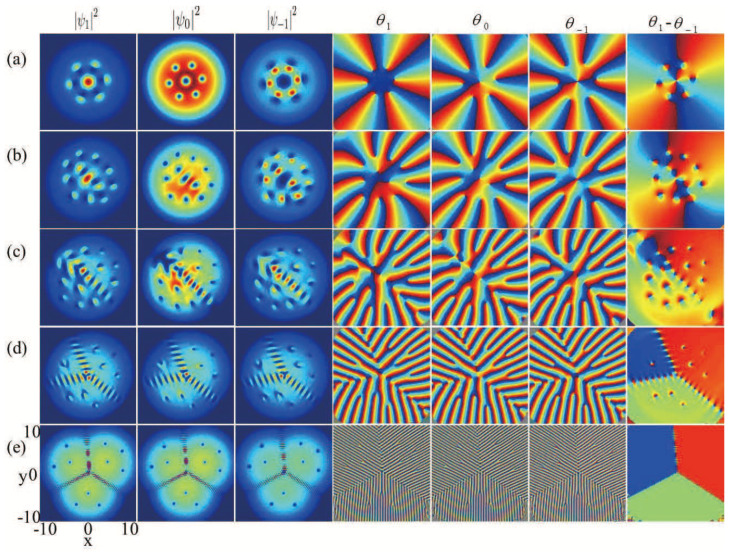
Effect of SU(3) SOC on the ground states of rotating spin-1 BECs with a fixed in-plane quadrupole field. The parameters are B=0.2,Ω=0.3, $\lambda_{0}$ =6000, and $\lambda_{2}=-50$. The first three columns denote the density distributions of the three components $|F=1,m_{F}\;=\;1\rangle$, $|F=1,m_{F}\;=0\rangle$, and $|F=1,m_{F}\;=-\;1\rangle$, and the corresponding phase distributions are given in the fourth to the sixth columns, respectively. The last column presents the phase difference of the two components $m_{F}\;=\pm 1$. The strengths of SU(3) SOC are given by (**a**) k=0, (**b**) k=0.2, (**c**) k=0.5, (**d**) k=1, and (**e**) k=5.

Then, we study the effect of SU(3) SOC in Spin-1 BECs under rotation with given quadrupole field strength B=0.2. The parameters are identical to those mentioned in Figure 4, with the exception of the strength of the SU(3) SOC. The density and phase distributions of the system’s ground states under different strengths of SU(3) SOC are displayed, where the fourth column represents the phase difference of components $m_{F}$ =±1. As depicted in Figure 6a, without SU(3) SOC (i.e., k=0), the distributions of component density and the arrangement of vortex structures exhibit regular distributions. The winding numbers in the central regions of BECs are $\langle 0,1,2\rangle ,$ with a dark soliton at the core area of the component $m_{F}$ =1. Thus, the vortex configuration in this system is an Anderson–Toulouse coreless vortice [43,44]. Additionally, each component contains six vortices surrounding the trap center, which evolve into a vortex necklace configuration in the azimuthal direction. The density distributions of the three components exhibit remarkable rotational symmetry. By calculating the phase difference between any two components (e.g., the phase difference of components $m_{F}$ =±1 given in the last column of Figure 6a), we find that six groups of the three-vortex structure cell are formed around the center of the condensates. With the increase of SU(3) SOC strength (see Figure 6b,c), the central Anderson–Toulouse coreless vortice disappears, but the number of vortices clearly increases. As shown in Figure 6c, a diagonalized serpentine chain forms; meanwhile, the other vortices are distributed as much as possible, beside the diagonalized serpentine chain (Figure 6c). When the SU(3) SOC further increases, the diagonalized serpentine vortex chain is transformed into a tree-fork-shaped vortex chain cluster, in which the vortices increase significantly. The distributions of density and phase in Figure 6d are similar to Figure 4b. For the case of strong SU(3) SOC strength k=5, three vortex chains that extend from the center of the BECs form an angle of $\frac{2\pi }{3}$ between any two components and divide the condensate into three parts. In the meantime, we observe that as the SU(3) spin–orbit coupling increases, the component densities of the BECs expand more significantly. This point can be understood. Essentially, when SU(3) SOC (with a given in-plane quadrupole field and the remaining parameters) increases, more angular momentum and energy contribute to the system and lead to the generation of more phase singularities and the expansion of the atom cloud. At the same time, the vortices away from the vortex chains are nearly synchronized (the last column of Figure 6e), therefore, the angular momentum and energy are carried by the vortices on the vortex chains (see Figure 6e).

Essentially, the in-plane quadrupole field exhibits a distinctive saddle point structure that tends to orient the spin inward toward the plane, which effectively suppresses the formation of ordinary vortices. In contrast, the rotation causes every component to generate vortices characterized by a single winding number. Conversely, to reduce the energy associated with the spin–exchange interaction, the ferromagnetic interaction encourages the spins to align in the identical direction. Because ordinary vortices are associated with a sudden change in the spin near their cores, the ferromagnetic spin–exchange interaction effectively inhibits the formation of these vortices [17]. In the absence of SU(3) SOC, the combined effect of an in-plane quadrupole field, rotation, and ferromagnetic spin interaction may result in the development of a regular density profile and vortex structure, as shown in Figure 6a. When the SU(3) SOC enhances vortex formation, the increasing vortices resulting from stronger SU(3) SOC are more likely to adopt a symmetrical equilibrium configuration.

**Figure 7:**
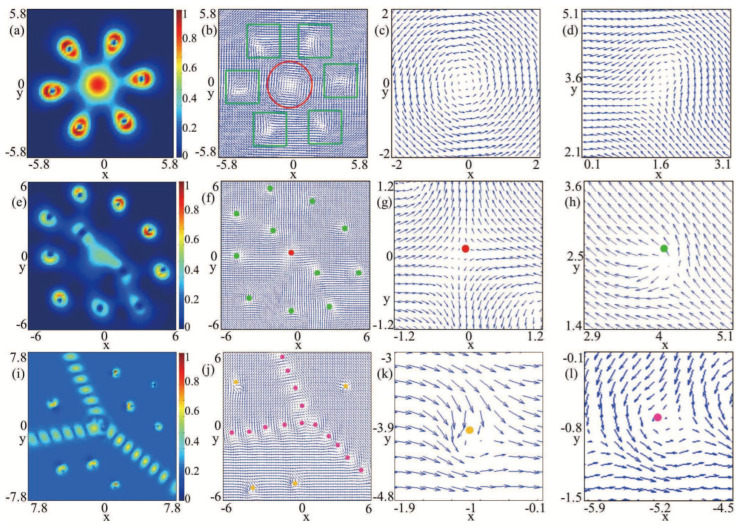
Spin structures. (**a**–**d**), (**e**–**h**), and (**i**–**l**) correlate to Figure 6a, Figure 6b, and Figure 6d, respectively. The topological charge densities are displayed in the 1st column, the spin textures are denoted in the 2nd column, and the local amplifications of the spin texture are presented by last two columns.

Now, to further elucidate the ground-state properties of Figure 6, we investigate the spin textures within the system. Displayed in Figure 7a is the topological charge density, and Figure 7b shows the corresponding spin texture. The ground states are given in Figure 6a. Figure 7c,d shows the local enlargements of the spin texture in Figure 7b. The results of our computations show that the topological excitation within the red circle pane corresponds to a circular skyrmion, characterized by a topological charge of Q=1, and those in every green square pane belong to a half-skyrmion, characterized by a topological charge of Q=0.5. Physically, the skyrmion highlighted within the red circular panel of the spin texture depicted in Figure 7b is associated with the Anderson–Toulouse coreless vortex located In the middle areas of the condensates, where the winding number in the middle areas of the three components is combined as $\langle 0,1,2\rangle$. In Figure 7b, the central circular skyrmion is encircled by six half-skyrmions in green square pane, which form a half-skyrmion necklace. So the topological structure of the system consists of an unusual skyrmion–half-skyrmion necklace, which is made up of a central skyrmion and an annular half-skyrmion (meron) necklace. As shown in Figure 7e,f, the topological charge density and corresponding spin texture are presented, respectively, which aligns with Figure 6b. Furthermore, Figure 7g,h shows the local amplifications of Figure 7f. In Figure 7f,g, the red spots represent hyperbolic skyrmions [39] with local topologial charge Q=1, which is related to the Anderson–Toulouse coreless vortex located in the middle area of the condensates. In Figure 7f–h, the local topological charge of every green dot is Q=0.5, which indicates that the system’s topological structure is a nearly symmetric half-skyrmion (meron) lattice in relation to the diagonal line composed of a half-skyrmion, hyperbolic skyrmion, and a half-skyrmion. In comparison, Figure 7i, Figure 7j, and Figure 7k,l display the topological density, spin texture, and local amplifications of the spin texture, where the ground state is displayed in Figure 6d. Considering the limited resolution of the texture, we only show the spin texture in the core region in Figure 7j, and the calculation results demonstrate that the topological defects in the rose red spots and yellow spots in Figure 7j–l are half-skyrmions with topological charge Q=0.5. Tree-fork-like half-skyrmions chains and half-skyrmions distributed between tree-fork vortex chains jointly form the topological configuration of this system. The spin textures correspond to the half-skyrmion in Figure 3, Figure 5 and Figure 7 and are related to the three-vortex structures with a winding configuration ${\left(1_{1},1_{0},1_{-1}\right)}_{3}$.

## 4. Conclusions

In summary, we have studied the in-plane quadrupole field-induced topological defects in SU(3) spin–orbit-coupled spin-1 BECs under rotation with ferromagnetic spin interactions, confined in a harmonic trap. Such a system sustains various types of topological structures and spin textures due to the multicomponent order parameters, in-plane gradient magnetic field, rotation, SU(3) SOC, and ferromagnetic spin interaction. When there is no rotation and the in-plane gradient magnetic field increases, we find the spin-1 BECs with SU(3) SOC experiences a transition from the initial Thomas–Fermi phase into the stripe phase, followed by a vortex–antivortex cluster state, and finally, a polar-core vortex state. In the absence of rotation but with a given quadrupole field, as the SU(3) SOC is enhanced, the system transitions from a central Mermin–Ho vortex into a vortex–antivortex cluster, and subsequently, it forms a bending vortex–antivortex chain. More notably, in the rotating situation, our research reveals the existence of five intriguing typical quantum phases as follows: a three-vortex-chain cluster with mutual angles of approximately $\frac{2\pi }{3}$, a tree-fork-like vortex chain cluster, a rotationally symmetric vortex necklace, a diagonal vortex chain cluster, and a density vortex cluster. Therefore, the in-plane quadrupole field, rotation, and SU(3) SOC, as important degrees of freedom, can be utilized to achieve the desired ground-state structures and regulate the phase transition among distinct ground states in spin-1 BECs. Furthermore, the system supports unique spin textures and skyrmion excitations, such as a bending half-skyrmion–half-antiskyrmion chain, three half-skyrmion (meron) chain with mutual angles of approximately $\frac{2\pi }{3}$, a slightly curved diagonal half-skyrmion (meron) cluster lattice, a skyrmion–half-skyrmion (skyrmion-meron) necklace, and a tree-fork-like half-skyrmion (meron) chain cluster lattice. These fascinating results have significantly deepened our understanding of topological defects and spin defects within ultracold atomic systems. Although implementing the current system in experiments may present challenges, it is theoretically feasible and can be achieved. For instance, one may consider a spin-1^87^Rb BEC [19,45] or a spin-1^23^Na BEC [46]. With the continuous advancement of cold-atom experimental techniques, it is anticipated that the system will be realized in the future, allowing for the observation of its novel quantum phases and dynamic properties experimentally.

## Data Availability

The original contributions presented in this study are included in the article. Further inquiries can be directed to the corresponding author.

## References

[1] Wu Z., Zhang L., Sun W., Xu X.T., Wang B.Z., Ji S.C., Pan J.W. (2016). Realization of Two-dimensional Spin-orbit Coupling for Bose–Einstein Condensates. Science.

[2] Yang S., Wu F., Yi W., Zhang P. (2019). Two-body Bound State of Ultracold Fermi Atoms with Two-dimensional Spin-orbit Coupling. Phys. Rev. A.

[3] Balasubramanian B., Manchanda P., Pahari R., Chen Z., Zhang W.Y., Valloppilly S.R., Li X.Z., Sarella A., Yue L.P., Ullah A. (2020). Chiral magnetism and high-temperature skyrmions in B20-ordered Co-Si. Phys. Rev. Lett..

[4] Lu Y.H., Wang B.Z., Liu X.J. (2020). Ideal Weyl Semimetal with 3D Spin-orbit Coupled Ultracold Quantum Gas. Sci. Bull..

[5] Wang Z.Y., Cheng X.C., Wang B.Z., Zhang J.Y., Lu Y.H., Yi C.R., Pan J.W. (2021). Realization of an Ideal Weyl Semimetal Band in a Quantum Gas with 3D Spin-orbit Coupling. Science.

[6] Cai Q.P., Zhang W.W., Lin L.W., Xu Y.G., Chen Z.X., Wang X.S., Yu H.P., Fang X.P., Zhang Y.C., Liu C.F. (2024). The Research Progress on One-Dimensional Spin-Orbit Coupled Fermi Gas. Prog. Phys..

[7] Gautam S., Adhikari S.K. (2018). Three-dimensional vortex-bright solitons in a spin–orbit-coupled spin-1 condensate. Phys. Rev. A.

[8] Ravisankar R., Sriraman T., Kumar R.K., Muruganandam P., Mishra P.K. (2021). Influence of Rashba spin–orbit and Rabi couplings on the spin-mixing and ground state phases of binary Bose–Einstein condensates. J. Phys. B.

[9] Pu D.D., Wang J.G., Song Y.F., Bai X.D. (2024). Magnetic supersolid phases of two-dimensional extended Bose-Hubbard model with spin–orbit coupling. New J. Phys..

[10] Sachdeva R., Tengstrand M.N., Reimann S.M. (2020). Self-bound supersolid stripe phase in binary Bose–Einstein condensates. Phys. Rev. A.

[11] Wang H., Wen L., Yang H., Shi C., Li J. (2017). Vortex states and spin textures of rotating spin–orbit-coupled Bose–Einstein condensates in a toroidal trap. J. Phys. B.

[12] Yang H., Gao Y., Yu B., Zhang J.H. (2022). Topological defects of dipolar bose-einstein condensates with dresselhaus spin–orbit coupling in an anharmonic trap. Front. Phys..

[13] Li Y., Liu Y., Fan Z., Pang W., Fu S., Malomed B.A. (2017). Two-dimensional dipolar gap solitons in free space with spin–orbit coupling. Phys. Rev. A.

[14] Wang Q., Zhao W., Wen L. (2021). Dynamics of rotating spin–orbit-coupled Bose–Einstein condensates in a quasicrystalline optical lattice. Results Phys..

[15] Xu X.Q., Han J.H. (2011). Spin-orbit coupled Bose–Einstein condensate under rotation. Phys. Rev. Lett..

[16] Yang H., Wang Q.B., Su N., Wen L.H. (2019). Topological excitations in rotating Bose–Einstein condensates with Rashba-Dresselhaus spin–orbit coupling in a two-dimensional optical lattice. Eur. Phys. J. Plus..

[17] Yang H., Su X.H., Zhang Y., Wen L.H. (2022). Topological excitations in rotating spin–orbit-coupled spin-1 Bose–Einstein condensates with in-plane gradient magnetic field. Commun. Theor. Phys..

[18] Liu C.F., Fan H., Zhang Y.C., Wang D.S., Liu W.M. (2012). Circular-hyperbolic skyrmion in rotating pseudo-spin-1/2 Bose–Einstein condensates with spin–orbit coupling. Phys. Rev. A.

[19] Campbell D.L., Price R.M., Putra A., Vald-Curiel A., Trypogeorgos D., Spielman I.B. (2016). Magnetic phases of spin-1 spin–orbit-coupled Bose gases. Nat. Commun..

[20] Graβ T., Chhajlany R.W., Muschik C.A., Lewenstein M. (2014). Spiral spin textures of a bosonic Mott insulator with SU(3) spin–orbit coupling. Phys. Rev. B.

[21] Han W., Zhang X.F., Song S.W., Saito H., Zhang W., Liu W.M., Zhang S.G. (2016). Double-quantum spin vortices in SU(3) spin–orbit-coupled Bose gases. Phys. Rev. A.

[22] Yue H.X., Liu Y.K. (2020). Composite solitons in SU(3) spin–orbit coupling Bose gases. Commun. Theor. Phys..

[23] Zhu Q.L., Shen J.M., Hua L., Liu F. (2022). Ground States of the SU(3) Spin–Orbit Coupled Bose–Einstein Condensate in a Rotating Annular Potential. J. Low Temp. Phys..

[24] Anderson B.M., Spielman I.B., Juzeliūnas G. (2013). Magnetically generated spin–orbit coupling for ultracold atoms. Phys. Rev. Lett..

[25] Ray M.W., Ruokokoski E., Tiurev K., Möttönen M., Hall D.S. (2015). Observation of isolated monopoles in a quantum field. Science.

[26] Tian L., Zheng N., Jian J., Liu W., Wu J., Li Y., Fu Y., Li P., Sovkov V., Ma J. (2022). Spin current in a spinor Bose–Einstein condensate induced by a gradient magnetic field. Chin. Phys. B.

[27] Aidelsburger M., Atala M., Lohse M., Barreiro J.T., Paredes B., Bloch I. (2013). Realization of the Hofstadter Hamiltonian with ultracold atoms in optical lattices. Phys. Rev. Lett..

[28] Kato M., Zhang X.F., Sasaki D., Saito H. (2016). Twisted spin vortices in a spin-1 Bose–Einstein condensate with Rashba spin–orbit coupling and dipole-dipole interaction. Phys. Rev. A.

[29] Arfken G.B., Webe H.J., Harris F.R. (2000). Mathematical Methods for Physicists.

[30] Mizushima T., Kobayashi N., Machida K. (2004). Coreless and singular vortex lattices in rotating spinor Bose–Einstein condensates. Phys. Rev. A.

[31] Ullah A., Balasubramanian B., Tiwari B., Giri B., Sellmyer D.J., Skomski R., Xu X. (2023). Topological spin textures and topological Hall effect in centrosymmetric magnetic nanoparticles. Phys. Rev. B.

[32] Wen L., Xiong H., Wu B. (2010). Hidden vortices in a Bose–Einstein condensate in a rotating double-well potential. Phys. Rev. A.

[33] Wen L., Li J. (2014). Structure and dynamics of a rotating superfluid Bose–Fermi mixture. Phys. Rev. A.

[34] Zhang Y., Mao L., Zhang C. (2012). Mean-field dynamics of spin–orbit coupled Bose–Einstein condensates. Phys. Rev. Lett..

[35] Su N., Wang Q., Hu J., Su X., Wen L. (2020). Topological defects in rotating spin–orbit-coupled dipolar spin-1 Bose–Einstein condensates. J. Phys. B.

[36] Tsubota M., Kasamatsu K., Ueda M. (2002). Vortex lattice formation in a rotating Bose–Einstein condensate. Phys. Rev. A.

[37] Mermin N.D., Ho T.L. (1976). Circulation and angular momentum in the a phase of superfluid Helium-3. Phys. Rev. Lett..

[38] Ullah A., Li X., Jin Y.L., Pahari R., Yue L., Xu X., Balasubramanian B., Sellmyer D.J., Skomski R. (2022). Topological phase transitions and Berry-phase hysteresis in exchange-coupled nanomagnets. Phys. Rev. B.

[39] Skyrme T.H.R. (1962). A unified field theory of mesons and baryons. Nucl. Phys..

[40] Schmeller A., Eisenstein J.P., Pfeiffer L.N., West K.W. (1995). Evidence for skyrmions and single spin flips in the integer quantized Hall effect. Phys. Rev. Lett..

[41] Aftalion A., Mason P. (2013). Phase diagrams and Thomas–Fermi estimates for spin–orbit-coupled Bose–Einstein condensates under rotation. Phys. Rev. A.

[42] Yang H., Zhang Q., Jian Z. (2022). Dynamics of rotating spin–orbit-coupled spin-1 Bose–Einstein condensates with in-plane gradient magnetic field in an anharmonic trap. Front. Phys..

[43] Liu C.F., Yu Y.M., Gou S.C., Liu W.M. (2013). Vortex chain in anisotropic spin–orbit-coupled spin-1 Bose–Einstein condensates. Phys. Rev. A.

[44] Mizushima T., Machida K., Kita T. (2002). Axisymmetric versus nonaxisymmetric vortices in spinor Bose–Einstein condensates. Phys. Rev. A.

[45] Luo X., Wu L., Chen J. (2016). Tunable atomic spin–orbit coupling synthesized with a modulating gradient magnetic field. Sci. Rep..

[46] Stenger J., Inouye S., Stamper-Kurn D.M. (1998). Spin domains in ground-state bose einstein condensates. Nature.

